# A mixed-methods evaluation of outreach service provision by the “Strengthening Migrant Access to Reproductive Health in Thailand” Initiative, 2020–2024

**DOI:** 10.3389/fgwh.2026.1637785

**Published:** 2026-04-10

**Authors:** Ahmar Hashmi, Ko Ko Aung, Nan San Wai, Prapatsorn Misa, May Myo Thwin, Kanyaw Paw, Suphak Nosten, Mi Wah Jitham, Chanapat Pateekhum, Waraporn Pimpasorn, Boonrid Wongchawengsup, Francois Nosten, Rose McGready

**Affiliations:** 1 Department of Nutrition Sciences and Health Behavior, School of Health Professions, University of Texas Medical Branch, Galveston, TX, United States; 2 Shoklo Malaria Research Unit, Mahidol-Oxford Tropical Medicine Research Unit, Faculty of Tropical Medicine, Mahidol University, Mae Ramat, Thailand; 3 Tak Public Health Office, Ministry of Public Health, Tak, Thailand; 4 Division of Public Health, Tak Provincial Administrative Office, Ministry of Interior, Tak, Thailand; 5 Centre for Tropical Medicine and Global Health, Nuffield Department of Medicine, University of Oxford, Oxford, United Kingdom

**Keywords:** family planning, implementation science, Myanmar, outcome and process assessment (healthcare), pregnancy, prenatal care, transients and migrants, vulnerable populations

## Abstract

**Introduction:**

Despite considerable progress, pregnancy-related health outcomes are still below Sustainable Development Goal targets for many low-to-middle-income countries. This study evaluated the Strengthening Migrant Access to Reproductive Health in Thailand (SMARH-T) Initiative that included an outreach service provision (2020–2024) to address upstream determinants of prenatal care and a family planning service provision for undocumented migrant women and newborns along the Thailand–Myanmar border.

**Methods:**

This study employed a sequential explanatory mixed-methods design with a quantitative survey followed by qualitative interviews and focus group discussions. Participants were asked about their experiences with the initiative and its delivery of prenatal and family planning services. Implementation outcome frameworks were used to understand the acceptability, end-user satisfaction, appropriateness, feasibility, reach, and sustainability of the initiative.

**Results:**

A total of 407 migrant women were surveyed and 17 interviews and discussions with health providers, staff, and stakeholders (*n* = 98) were conducted. The outreach service provision allowed for comparable convenience (*p* < 0.001), travel time (<30 min, *p* < 0.001), and costs (<USD 2.75, *p* < 0.001) to reach care compared with women receiving services at fixed clinics. A thematic analysis of qualitative data demonstrates the acceptability, appropriateness, and improved reach due to the outreach service provision, despite the logistical and management burden involved. Improved organizational processes for program logistics and administration helped enhance the sustainability of the initiative.

**Discussion:**

This mixed-methods evaluation of the SMARH-T Initiative demonstrated high service utilization, satisfaction, and reach for migrant women along the Thailand–Myanmar border. The multipronged approach incorporated reparative strategies to address this particularly vulnerable population in this context.

## 1 Introduction

Despite a considerable reduction in maternal and neonatal mortality and morbidity worldwide, progress still needs to be made on this front. Global maternal mortality rates show a preventable loss of nearly 3 million women between 2010 and 2020 (1). There has been a reduction in maternal mortality rates between 2000 and 2020 in the Southeast Asia region, with the rates dropping from 231 to 134 deaths per 100,000 live births (2). However, this far exceeds the Sustainable Development Goal of 70 deaths per 100,000 live births.

Greater improvements to maternal mortality rates have yet to be fully realized, but there is considerable evidence that efficacious interventions exist. There is evidence dating back to the early 2010s that recommends life-saving interventions from preconception to up to 2 years of life that are predominantly curative but also include preventive measures (3–6). Some interventions involve the community to improve pregnancy, postnatal, and family planning outcomes, such as community or lay health workers who shift tasks away from facility-based care (7–9), community health education (10), and mHealth interventions (11).

Interventions may be tested singly or as components of a program, but as Souza et al. claim, many do not break from the biomedical mold with their focus on determinants of hospital-based care (1, 12). However, effective programming that improves maternal mortality, morbidity, intrapartum, and newborn outcomes requires multipronged approaches that would better address structural and social determinants (1). Programming that does not address structural and social determinants may limit the potential of proven interventions to improve pregnancy-related health outcomes.

Beginning in 2020 (13), the Strengthening Migrant Access to Reproductive Health in Thailand (SMARH-T) Initiative brought together a package of interventions and implementation strategies for improved community-based provision of prenatal care and family planning services for migrant women along the Thailand–Myanmar border. In line with global and Southeast Asian trends, this largely undocumented migrant population has not seen a great fall in maternal mortality rates that would be needed to reach the Sustainable Development Goals. Among migrant women in these communities, vulnerability and intersectionality play out in a number of ways: costly and difficult transportation over difficult terrain (13–20), a lack of community and/or health infrastructure (15, 19–25), the stigmatization and marginalization of the migrant poor (14, 19, 20, 23–30), and sociocultural and language barriers that lead to low health literacy and awareness of reproductive health needs in migrant communities along the border (15, 31–36).

Therefore, the SMARH-T initiative was designed and implemented to address the structural and social barriers experienced by this population in accessing prenatal care and family planning. This paper is a mixed-methods evaluation exploring the acceptability, appropriateness, feasibility, reach, and sustainability of this program from the perspective of migrant women, providers, community health workers, and partners participating in the SMARH-T Initiative.

## 2 Methods

### 2.1 Study design and participants

This study employed a sequential explanatory mixed-methods design with quantitative surveys (October 2023) followed by qualitative in-depth interviews and focus group discussions (November to December 2023). Participants in the survey included women (≥18 years of age) who were receiving prenatal care or seeking family planning services at fixed clinics or at outreach sites. Participants in the qualitative component included only doctors, medics, nurses, and staff directly involved in the service provision at fixed clinics and outreach sites. Interviews and discussions also included participants not directly providing care services such as community health workers (Aw Sor Tho) and reproductive health stakeholders within the NGO, private, and Thailand public health sectors.

### 2.2 Setting

This study took place in Tak Province, Thailand, along the Thailand–Myanmar border, and was conducted by the Shoklo Malaria Research Unit (SMRU) and the Borderland Health Foundation (BHF). SMRU is headquartered in Tak Province, Thailand, and is a field research station for the Mahidol–Oxford Tropical Medicine Research Unit within the Faculty of Tropical Medicine at Mahidol University (37). Between 1986 and 2017, SMRU provided health programming for refugees (“persons fleeing violence”) in refugee camps in Thailand (22). In 1998, SMRU established clinics to serve economic migrant communities along the Thailand–Myanmar border as well (22). Although SMRU operations in refugee camps have closed, they are still ongoing in migrant communities. BHF was registered as a humanitarian foundation in Thailand in 2019 to operationalize and implement health and humanitarian programming that SMRU had begun, including the administration, logistics, and management of prenatal and family planning service delivery.

SMRU/BHF provides services free of charge for migrant women in the rural areas of Tak Province. Migrants in this region are non-Thai and originally from neighboring Myanmar. These groups are predominantly of Karen or Burmese ethnicity (38). Although migrating to the border region seeking economic opportunities, most migrants seeking health services at SMRU/BHF are undocumented and therefore have restricted access to comprehensive prenatal, birthing, and family planning services provided in the Thai public health sector. The total catchment of SMRU/BHF clinics is estimated at about 200,000 undocumented migrants in this region, and fixed clinics perform approximately 2,500 deliveries every year (22).

**Figure 1:**
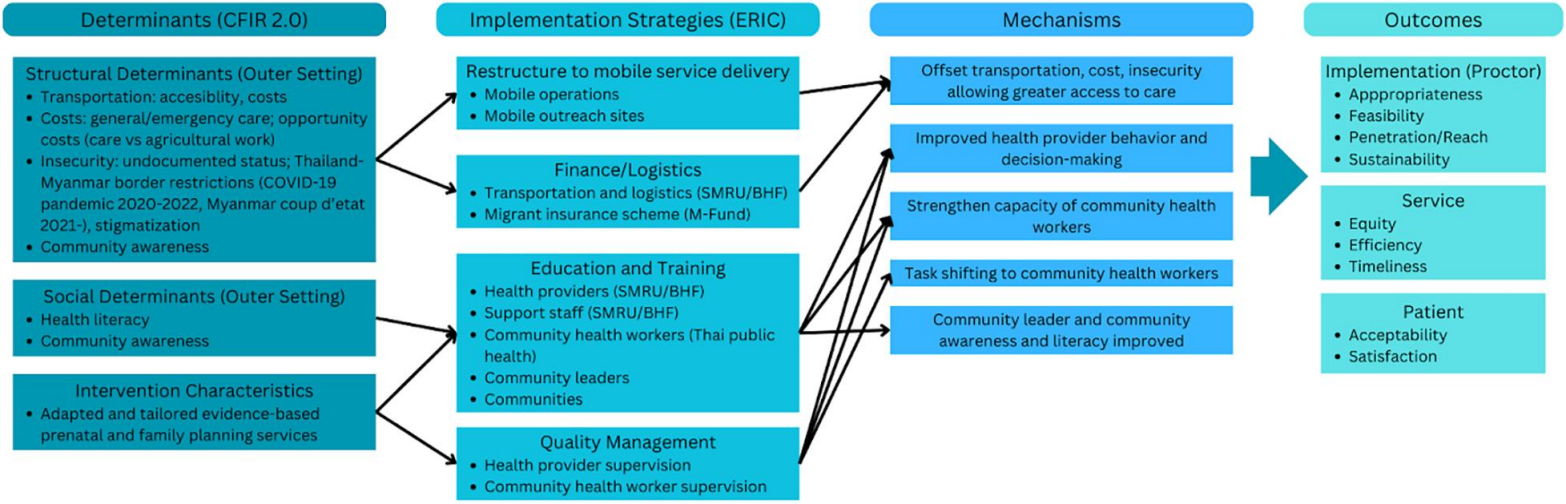
An implementation logic model for a package of implementation strategies (68) in establishing an outreach model of prenatal care and family planning services. Adapted from Smith et al. (69).

Migrant women along the border often have lower socioeconomic status and limited formal education than Thai residents (21, 31, 32). When seeking reproductive health services in Thailand, migrant women face discrimination and security concerns given their undocumented status (14, 23, 25, 27, 30). This is further exacerbated by issues with transportation costs associated with prenatal care follow-up, delivery, and family planning (13, 15, 18–20). The outreach program was developed and designed with this in mind, where the central logic of the program was to provide easier access to reproductive health services on outreach to offset these social and structural determinants affecting migrant women and newborn health outcomes (Figure 1).

Beginning in 2020, SMRU/BHF began an outreach program to improve delivery and utilization of prenatal and family planning services among non-Thai migrant women in rural Tak Province, Thailand. This program was designed to provide as many prenatal care and family planning services as possible at outreach sites within community spaces to enhance migrant women access to these services. Outreach teams operated out of the two SMRU/BHF fixed clinics in Maw Ker Thai (Phop Phra district, Tak) and Wang Pha (Mae Sot/Mae Ramat districts, Tak) and were staffed by doctors, midwives, medics, nurses, ultrasonographers, laboratory technicians, and IT staff. This cadre of health providers was able to provide comprehensive prenatal and family planning services on outreach, except for birthing, which occurred at the labor and delivery wards at SMRU/BHF fixed clinics. The staff often come from the same communities as the patients, are able to speak local languages, and appreciate local cultural norms.

**Figure 2:**
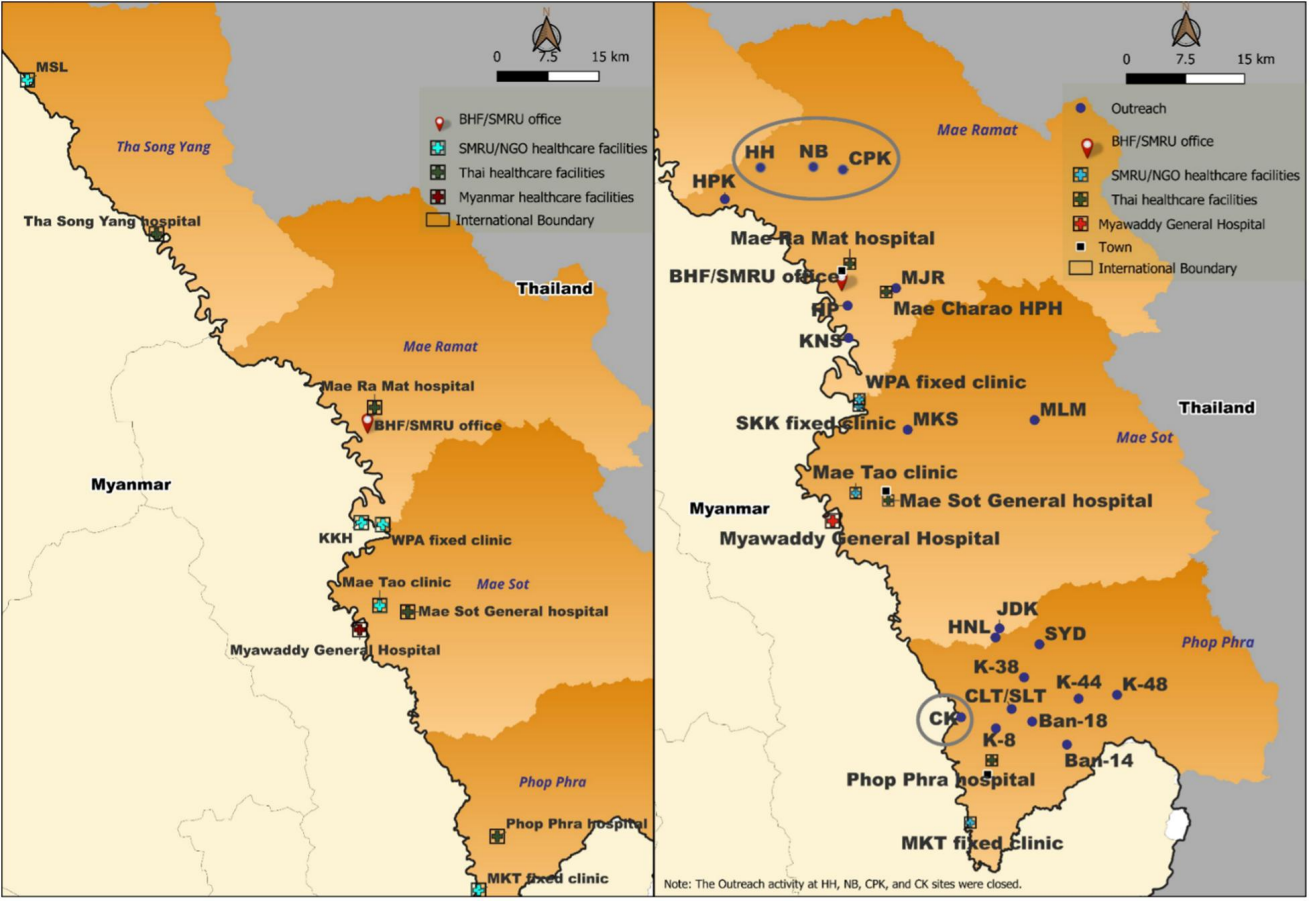
Outreach sites and geographic distribution across the Thailand–Myanmar border. L, prior to outreach; R, outreach sites by study end.

The outreach program was set up in two phases—a pilot phase followed by a scale-up phase. In the pilot phase (2021–2022), two outreach sites were selected for each fixed clinic in two districts of Tak Province for a total of four sites. By the end of the scale-up phase (2022–2023), outreach teams provided services in 13 sites across the two districts (Figure 2). Outreach visits were conducted 4 days a week, with each outreach site visited once every 2 weeks.

Evidence-based practice for prenatal and family planning services provided on outreach visits aligns with the 2016 WHO recommendations on antenatal care (4) and the 2022 edition of the WHO family planning handbook (5) (Supplementary Table S1). The roles and responsibilities of Aw Sor Tho are also outlined in Supplementary Table S1. Where applicable, services have been adapted and tailored to the context through operational research and regular local monitoring of best, safe practices by SMRU/BHF (3, 16). For example, gestational diabetes screening is selective instead of comprehensive for all women given its cost constraints in this setting.

### 2.3 Quantitative and qualitative study components

#### 2.3.1 Conceptual framework and research paradigm

Conceptually, this study aligns well with the “vulnerability trajectory” outlined by Sheikh et al. where the outreach program as a whole provided several reparative strategies to improve migrant maternal and newborn health (6). Specifically, the “intervention” in question could be viewed as the outreach program as a whole and/or specific prenatal and/or family planning services provided on outreach. As such, this study seeks to understand how the implementation of the outreach program acts as a set of reparative strategies to address the vulnerabilities of migrant women while navigating their reproductive health. Figure 1 shows the implementation research logic model used to frame the quantitative and qualitative results of this study, highlighting the determinants of prenatal and family planning service provision, mapping the implementation strategies used in the SMARH-T program to the Expert Recommendations for Implementing Change (ERIC), the mechanisms by which the implementation strategies address the determinants, and the implementation outcomes.

To do this, the study explored the perspectives of women of reproductive age receiving BHF/SMRU services and key stakeholders (outreach staff and stakeholders from NGO, private, and public sectors) involved with this outreach program. The study analysis aligned these mixed-methods components (quantitative survey and qualitative interviews/discussions) to understand participant perspectives with respect to the outreach program and/or the prenatal and family planning services provided. Proctor's implementation outcomes were identified as a framework to be used in this study given the implementation focus of this outreach program (39, 40). Using this framework deductively to guide the analysis, the research team reviewed and discussed the Proctor definitions for outcomes and reached a consensus on qualitatively determining the outcomes most appropriate to this work: acceptability, appropriateness, feasibility, penetration or reach, and sustainability. These outcomes were chosen as they provided more pertinent data for a review of a program nearing completion. Table 1 highlights the study implementation outcomes of interest, their definitions, and the operationalized definitions used for deductive coding and thematic analysis (41). Finally, the analysis followed a constructivist paradigm (42), allowing participants to express their views as a valid, lived reality. The qualitative components were designed to compare, contrast, triangulate, and corroborate information from the quantitative survey.

**Table 1:** Implementation outcomes of interest, definitions, and operationalized definitions for qualitative study components.

Implementation outcome	Definition	Operationalized definition
Acceptability	The belief that an intervention is agreeable, palatable, or satisfactory	The outreach program and prenatal and family planning services are acceptable to implementers, stakeholders, and/or migrant women
Appropriateness	A perceived fit, relevance, and compatibility of an intervention to a given context or a perceived fit for a problem	The outreach program and/or its services is/are perceived as a good fit in meeting the MNCH needs of migrant women
Feasibility	The extent to which an implementation target is successful or deployed in a given setting	Participants provide information about barriers and/or enablers for outreach program implementation
Penetration or reach	The saturation of an intervention within a service setting	The number of women receiving prenatal and/or family planning care through outreach visits
Sustainability	The extent to which an implementation target is maintained or institutionalized within a service setting	Participants offer insight into barriers and/or enablers that helps maintain the outreach program and/or institutionalize the key elements of the program within the SMRU/BHF and among partners

#### 2.3.2 Research team

The research team was involved in all aspects of the evaluation, from study protocol design to analysis. RM, PM, CP, and AH were responsible for study conceptualization and design. The team members responsible for designing the quantitative and qualitative components of this study were outreach support staff with a background in social science research (PM, KKA, MM, SN, and KP). This team mirrored the diversity of the participants and included Thai, Burmese, and Karen members. These team members were not directly responsible for the services received by migrant women at SMRU/BHF.

#### 2.3.3 Survey

A survey instrument was developed to understand how migrant women perceived prenatal or family planning services received at outreach sites compared with fixed clinics. The survey instrument was informed by the REPRO-Q survey tool (43), to capture outreach site access, service satisfaction, respect, and privacy. Surveys were conducted as one-to-one interviews between study team members and migrant women in their preferred language. The team members then collated the survey answers, which were compiled in Microsoft Excel.

Sample size calculations were performed prior to study commencement. For prenatal care, the rate of satisfaction was expected to be high at 80% for fixed clinic care, while it was estimated to be lower at 60% for outreach clinics due to the limited number of outreach days at a given site and fewer available services. Assuming a power of 80% and an alpha of 5%, the sample size was calculated at 91 in each group to detect a difference. For family planning, the satisfaction rate for outreach sites was expected to be higher at 90%. Assuming a power of 90% and an alpha of 5%, a sample size of *n* = 98 was determined to detect a difference. Therefore, stratified random sampling for the survey was intended for a total of 200 migrant women for prenatal services and for 200 migrant women for family planning services, divided equally among women presenting to the fixed clinic and outreach sites for these services, and equally across the two districts (*N* = 400).

#### 2.3.4 Interviews and focus group discussions

Interviews and focus group discussions were developed to understand the perceptions of stakeholders directly or indirectly involved in the outreach program. Team members facilitated these discussions and followed semi-structured guides aligned with Proctor's implementation outcome constructs of acceptability, appropriateness, feasibility, reach, and sustainability of prenatal care and family planning services provided on outreach.

Participants were selected using a stratified opportunistic sampling strategy, stratified according to outreach site to ensure representation from each. Prior to beginning interviews/discussions, all study team members met to review the semi-structured guides and to reach a consensus on how the guidelines would be operationalized. All interviews and discussions were conducted by at least two facilitators from the study team. One facilitator led the discussion in the preferred language of the participant(s), with another facilitator taking notes. All interviews and discussions were audio-recorded. Following all interviews and discussions, the facilitators debriefed with AH and the team members to identify the key themes emerging from each respective discussion. At these debriefs, saturation was assessed until it was met. All notes were compiled with summaries of the debriefings.

### 2.4 Analysis

#### 2.4.1 Survey

All outcomes were analyzed according to site where services were received, that is, the fixed clinic or the outreach site, as the independent variable. All outcomes were analyzed separately for women receiving only prenatal care and those receiving only family planning services.

Outcomes of interest from survey data included estimated cost, total travel time, and perceived convenience in accessing the care site. In addition, outcomes from the survey included women's perceived satisfaction, privacy, and respect for the services received on outreach. Outcomes were converted to categorical data for costs and total travel time. Costs were divided into three categories: <100 Thai Baht (<USD $2.75), 101–200 Thai Baht (USD $2.76–$5.50), and >200 Thai Baht (>USD $5.50). Total travel time was categorized as <30, 31–60, or >60 min. Travel stops were categorized as no stops, 1 stop, or >1 stop requiring a change in transportation mode. Women subjectively reported whether access to care site was convenient (“yes/no”). Women satisfaction, privacy, and respect were categorized as “satisfied” or “dissatisfied” or “yes” or “no.” A subset of survey data was analyzed for women's “second choice” option for receiving care besides the fixed clinic or outreach sites. Women's perceived safety and security concerns in accessing service delivery sites were elicited as open-ended responses during the survey and were categorized according to main themes. The proportion of women's responses regarding their second choice of prenatal services after fixed clinics and outreach sites were presented in a grouped bar chart, while their primary concerns in accessing prenatal care and about the impact of crises on family planning access were shown as pie charts.

Continuous data were summarized using mean, standard deviation, minimum, and maximum values. Categorical data were summarized using counts and percentages. Non-parametric tests (Fisher's exact test) were used to compare women's responses to services received at fixed clinics and outreach sites (singly). *T*-tests were used to compare differences between groups. Corrections were applied when comparing a single dataset with many other datasets or performing numerous tests on one dataset, such as multiple *t*-tests or *post hoc* analyses after an ANOVA. All reported *p*-values were two-sided, and *p*-values <0.05 were considered statistically significant. All analyses were performed using Stata version 18 (StataCorp, College Station, TX, USA).

#### 2.4.2 Interviews and focus group discussions

Following the completion of all interviews and focus group discussions, the team (KA, MT, SN, and KP) conducted deductive qualitative analysis. Proctor's implementation outcomes of accessibility, appropriateness, feasibility, reach, and sustainability were operationalized and a codebook with definitions of these key concepts was developed through consensus (Table 1). KA, MT, SN, and KP then began deductive coding based around Proctor's implementation outcomes, analyzing the interviews/discussions that these team members conducted (2–3 interviews/discussions each). The team collated their analysis according to the implementation outcome codes and met to discuss any disagreements. Once a consensus was obtained, implementation outcome codes with data from interviews/discussions were compiled into a Microsoft Excel file and shared with AH. AH then performed thematic analysis and confirmed findings with KA, MT, SN, and KP. Results were drafted and all team members reviewed the methods and analysis results to confirm their fidelity to, corroboration, and triangulation of the qualitative data collected.

## 3 Results

### 3.1 Survey

A total of 407 women were surveyed: 204 were surveyed when reporting for prenatal care (Table 2) and 203 when reporting for family planning services (Table 3). A total of 154 women were surveyed at fixed clinics and 253 at outreach sites. Women reporting to either fixed clinics or outreach or prenatal care/family planning totaled 274 in Phop Phra and 133 in Mae Ramat districts, respectively (Tables 2, 3). The median age of the participants was 24 years (IQR 20-30.8) with a median gravidity of 2 (IQR 1–2). As expected, there was a general trend of perceived convenience at the outreach sites (Tables 2, 3), likely due to appropriate site selection for outreach activities.

**Table 2:** Demographics of migrant women (*n* = 204) attending prenatal services at fixed clinics or outreach sites.

Variables		MRM district (*n* = 67)			PP Sites (*n* = 137)		
		Fixed (*n* = 37)	Outreach (*n* = 30)	*P*-value	Fixed (*n* = 70)	Outreach (*n* = 67)	*P*-value
Age, year, median	[IQR] (Min–Max)^a^	24 [22–30] (16–42)	26 [20–30] (17–40)	0.699	23 [19–31] (15–43)	24 [20–31] (17–42)	0.167
Gravidity, median	[IQR] (Min–Max)^a^	1 [1–2] (1–5)	1 [1–3] (1–7)	0.554	2 [1–3] (1–6)	2 [1–2] (1–7)	0.222
Language/ethnicity (*n* = 203)^b^	Karen^c^	2 (6.67)	2 (5.4)		37 (52.9)	0 (0)**	
	Burman	35 (94.6)	27 (90.0)		32 (45.7)	66 (98.5)**	
	Other^d^	0 (0)	1 (3.3)	0.518	1 (1.4)	1 (1.5)	<0.001
Transportation costs^b^^,^^e^	< USD $2.75	36 (97.3)	24 (80.0)*	0.048	60 (85.7)	58 (86.6)	0.358
	USD $2.75–5.50	0 (0)	4 (13.3)*		6 (8.6	8 (11.9)	
	≥ USD $5.50	1 (2.7)	2 (6.7)		4 (5.7)	1 (1.5)	
Travel time^b^	<30 min	25 (67.6)	13 (43.3)	0.054	27 (36.6)	36 (53.7)*	0.203
	30–59 min	11 (29.7)	12 (40.0)		27 (38.6)	20 (29.9)	
	≥60 min	1 (2.7)	5 (16.7)		16 (22.9)	11 (16.4)	
Convenient to access (*n* = 203)^b^	Yes	36 (97.3)	29 (96.7)	1.000	60 (85.7)	65 (98.5)	0.009
	No	1 (2.7)	1 (3.3)		10 (14.3)	1 (1.5)**	
Privacy (*n* = 203)^b^	Satisfied	36 (97.3)	30 (100)	1.000	69 (98.6)	67 (100)	1.000
	Dissatisfied	1 (2.7)	0 (0)		1 (1.4)	0 (0)	
Respect (*n* = 202)^b^	Satisfied	36 (100)	29 (100)	1.000	36 (100)	67 (100)	n.a.
	Dissatisfied	0 (0)	0 (0)		0 (0)	0 (0)	
Discrimination (*n* = 200)^b^	No	36 (07.3)	30 (100.0)	1.000	69 (98.6)	67 (100)	1.000
	Yes	1 (2.7)	0 (0)		1 (1.4)	0 (0)	
Overall Satisfaction (*n* = 200)^b^	Yes	37 (100)	28 (100)	n.a.	67 (95.7)	67 (100)	0.245
	No	0 (0)	0 (0)		3 (4.3)	0 (0)	

MRM, Mae Ramat district; PP, Phop Phra district.

^a^ Mann–Whitney *U* test comparison medians.

^b^ Chi-Squared test or Fisher's Exact Test (count <5), Bonferroni correction.

^c^ “Karen” includes Sgaw and Pwo Karen.

^d^ “Other” includes Mon.

^e^ Cost conversion to Thai Baht (THB): < USD $2.75 = < THB 100; USD $2.75–5.50 = THB 100–199; ≥ USD $5.50 = ≥ THB 200.

* *p* < 0.05.

** *p* < 0.001.

**Table 3:** Survey responses of migrant women (*n* = 203) for family planning services received comparing fixed clinics with outreach sites in each district.

Variables	MRM district (*n* = 65)			PP district (*n* = 138)		
	Fixed (*n* = 31)	Outreach (*n* = 34)	*P*-value	Fixed (*n* = 23)	Outreach (*n* = 115)	*P*-value
Age, y, Median [IQR] (Min-Max)^a^	32 [23–35] (18–45)	26 [21–33] (17–48)	0.255	27 [23–34] (18–39)	26 [21–33] (14–45)	0.368
Gravidity, Median [IQR] (Min-Max)^a^	2 [1–3] (1–6)	1 [1–3] (0–6)	0.060	2 [1–3] (0–6)	2 [1–2] (0–10)	0.106
Language/Ethnicity (*n* = 203)^b^						
Karen^c^	6 (19.4)	1 (2.9)	0.053	7 (30.4)	2 (1.7)*	<0.001
Burman	24 (77.4)	33 (97.1)		16 (69.6)	112 (97.4)*	
Other^d^	1 (3.2)	0 (0)		0 (0)	1 (0.9)	
Convenient to access (*n* = 203)^b^						
Yes	29 (93.5)	33 (97.1)	0.602	22 (95.7)	113 (98.3)	0.424
No	2 (6.5)	1 (2.9)		1 (4.3)	2 (1.7)	
Discrimination (*n* = 203)^b^						
No	31 (100)	34 (100)	n.a.	22 (100)	112 (97.4)	1.000
Yes	0 (0)	0 (0)		0 (0)	3 (2.6)	
Overall Satisfaction (*n* = 203)^b^						
Yes	31 (100)	34 (100)	n.a.	22 (95.7)	114 (99.1)	0.307
No	0 (0)	0 (0)		1 (4.3)	1 (0.9)	

n.a., not applicable; MRM, Mae Ramat District; PP, Phop Phra District.

^a^ Mann–Whitney *U* test comparison medians.

^b^ Chi-Squared test or Fisher's Exact Test (count <5), Bonferroni correction.

^c^ “Karen” includes Sgaw and Pwo Karen.

^d^ “Other” includes Rakhine or Mon.

* *p* < 0.001.

Tables 2, 3 summarize additional demographics and patient self-report of satisfaction, privacy, and respect during their visit. The majority of women presenting for prenatal care or family planning services mentioned that sites were convenient to access (190/204, 93.1%), with women attending for prenatal care spending <USD $2.75 (THB 100; 181/204 women, 88.7%) and requiring less than 30 min (108/204, 52.9%) to reach care. Women had few concerns on matters of privacy, respect, or facing discrimination, with most of them reporting being overall satisfied with their visit for prenatal care or family planning services (398/403, 98.8%). For disaggregated data by district, please refer to Tables 2, 3.

**Figure 3:**
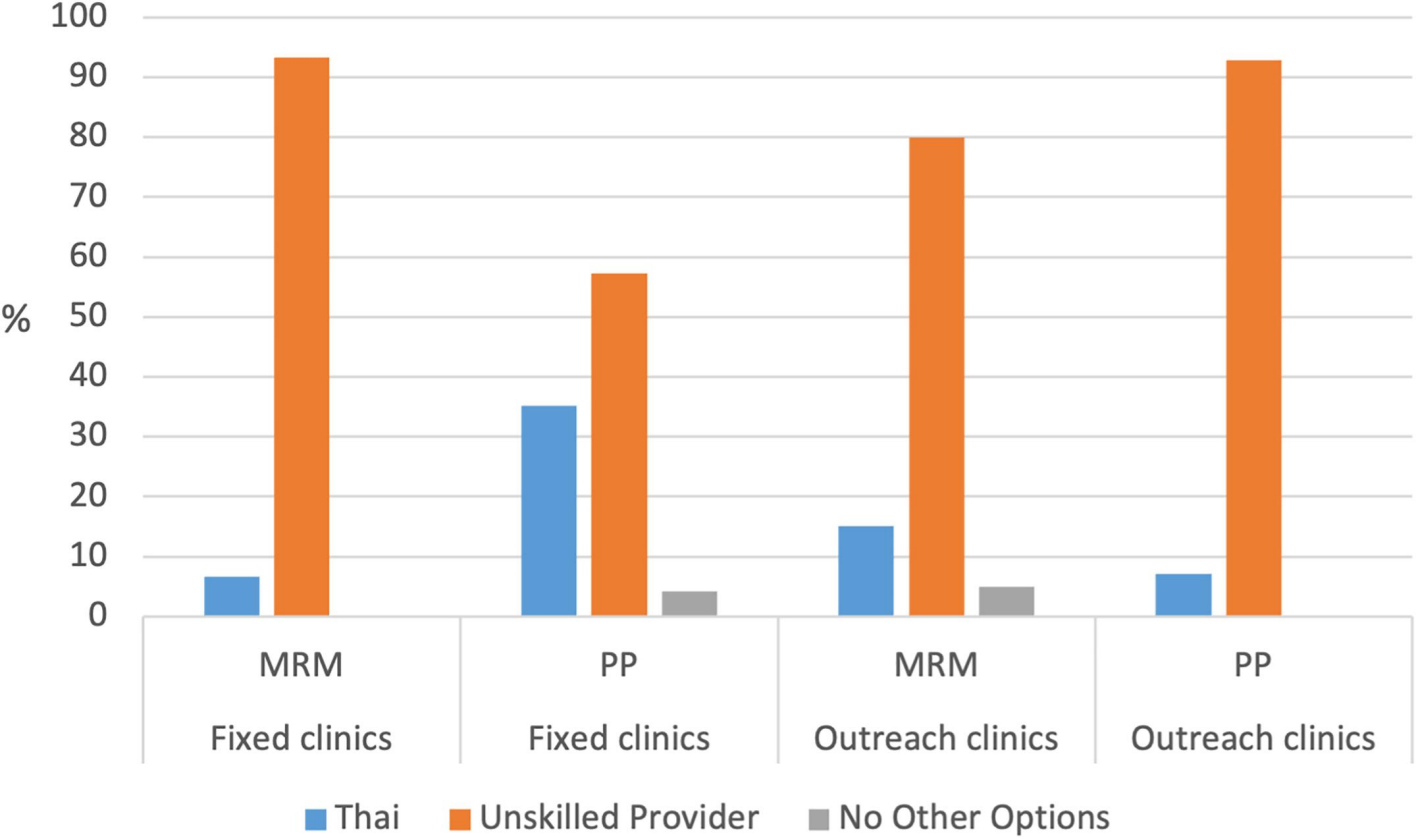
Women who were interviewed at a fixed clinic or an outreach site (i.e., their first choice) describe their second choice (%) for prenatal care services. MRM, Mae Ramat District; PP, Phop Phra District. Significant for all fixed compared with all outreach clinics and comparing MRM with PP (*p* < 0.001).

**Figure 4:**
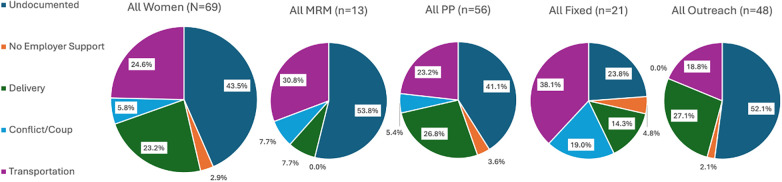
Most cited concerns that migrant women have in accessing prenatal services, for all women (*n* = 69) and disaggregated by district and fixed clinics/outreach sites. MRM, Mae Ramat District; PP, Phop Phra District.

Additional findings from the survey include women's choice for services and disruption of care due to the COVID-19 pandemic and/or the 2021 coup in Myanmar. Figure 3 shows women's second choice for receiving prenatal care if they could not receive care at SMRU/BHF fixed clinics or outreach sites. According to the 163 women who provided information about their second choice after fixed clinics and outreach sites, 77.3% (126/163) would seek the services of unskilled birth attendants or providers at home as an alternative (*p* < 0.001 for PP vs. MRM and fixed vs. outreach). Only 2.5% (4/163) of women reported that they would have no alternative for prenatal care. Figure 4 highlights women's concerns in accessing prenatal services, with 26.5% (69/204) expressing a concern, and the primary concerns reported across all sites were (1) insecurity due to their undocumented status (30/69, 43.5%); (2) transportation (17/69, 24.6%); (3) safe delivery attended by trained professionals in a sterile environment (16/69, 23.2%), and (4) a lack of employer support (5/54, 9.3%). Notably, only 5.8% (4/69) of women reported concerns due to the coup in Myanmar. Of all women presenting for family planning services, only 23/203 (11.3%) mentioned access disruption due to the COVID-19 pandemic and/or the Myanmar coup; 20/23 of these women were from PP district; and 10/23 of these women specifically linked their prior pregnancy to disruption of access to family planning services.

### 3.2 Qualitative results

A total of 17 interviews and discussions were completed between November and December 2023. A total of 98 participants (66 women and 32 men) participated in three in-depth interviews (*n* = 3) and 14 focus group discussions (*n* = 95). Participant age ranged from 20 to 65 years. A broad range of stakeholders involved in migrant maternal, newborn, and child health along the Thailand–Myanmar border participated, including SMRU/BHF staff (doctors, medics, nurses, midwives, ultrasonographers, health assistants, household workers, IT staff, and program support staff); migrant community health workers (Aw Sor Tho) working closely with SMRU/BHF on the implementation of outreach services; and Thai health officials.

#### 3.2.1 “The results speak for themselves”

In general, those participating in the qualitative components of this study deemed the outreach program a success. Across all participants, the program was viewed as effective in reaching migrant communities and providing women with high-quality care and addressing their unmet needs for prenatal care and family planning. This explained the survey findings across many women and the breadth of services that they were more conveniently able to access.

Importantly, in praising this program, the participants also validated the underlying logic of the design of outreach activities in addressing the social and structural determinants of the health of migrant women (Figure 1). These aligned with women's main concerns for accessing prenatal care in the surveys: insecurity due to lack of documentation, transportation concerns, and costs associated with accessing fixed clinics. The qualitative findings corroborate the quantitative surveys, where women report greater convenience in access to outreach sites, lower costs, and lower anxiety in moving about the border region without documents.

Women don't need to go very far and don't have to fear the legal documents, and it drops the cost for transportation a *lot* [emphasized by participant]. They are worried that if the outreach sites are not there, most people will not even seek services at the fixed clinic, and it will lead to more teenage pregnancies, more pregnancies, more TOP [termination of pregnancy services].—Focus group discussion with Aw Sor Tho, Mae Ramat District.

Now the distance reached by services provided have increased and places that they [women] have to travel has decreased. The patients seeking help from TBAs [traditional birth attendants] are also referred to outreach [SMRU/BHF].— Focus group discussion with Aw Sor Tho, Phop Phra District.

The participants pointed to direct benefits to the health of migrant women that they observed: earlier initiation of prenatal care, fewer pregnancy complications, improved follow-up during the prenatal period, enhanced utilization of family planning services, fewer obstetrical emergencies, and improved provision of prenatal and family planning services for adolescents.

Situated against a backdrop of the COVID-19 pandemic and the coup in neighboring Myanmar, the participants claimed that this program readily addressed the unmet reproductive health needs of migrant women. Indirect benefits included the following: increasing the capacity of SMRU/BHF staff and Aw Sor Tho in providing quality, sensitive, and comprehensive care; community engagement leading to improved community awareness around prenatal, delivery, and family planning needs; changing provider perceptions of the quotidian experiences of migrant women; and encouraging the acceptance of family planning counseling and service provision. Participant recommendations for improving this program focused on expanding the number of outreach sites and providing primary care services for adults with chronic diseases, for example.

The qualitative results highlight specific themes related to Proctor's outcomes, grouped as outlined by the participants (Table 1): (1) acceptability and appropriateness and (2) reach (or penetration), feasibility, and sustainability. Participant views about the acceptability and appropriateness of the program were often weighed against issues concerning reach, feasibility, and sustainability, providing deep insight into the impact of this program and corroborating key findings from the surveys of migrant women receiving services.

#### 3.2.2 Acceptable and appropriate services

Aligned with satisfaction, ease of access, and the number of services that women sought at outreach sites, the participants in the interviews and discussions provided more details on the appropriateness of the program in meeting women's needs:

- Improving MNCH outcomes through early and comprehensive prenatal care and utilization of family planning services; reduced obstetrical emergencies; and higher rates of adolescents receiving care;
- Offsetting the social and structural determinants preventing migrant women from accessing timely and quality care;
- Training activities providing Aw Sor Tho with actionable information while strengthening their skills, confidence, and competence; and
- Community engagement activities that improved community awareness and demand for MNCH services.

The outreach program as a whole and the services offered were viewed as acceptable to migrant women and the community at large. The combination of an acceptable and appropriate outreach program led to the success observed by the participants. These two outcomes were often discussed together.

There are no prenatal services from other organizations like the kind SMRU/BHF provide. It is good that SMRU/BHF come into marginal communities. In the past people got sick or were about to deliver a baby and died on the way to the hospital.—Interview with Thai village leader.

From the [Thai Health Promoting Hospital in the Thai Public Health system] perspective, the outreach project is successful because it is not passive as it goes into the community and provides for migrant needs.— Focus group discussion with Thai health officers.

Importantly, the participants' assessments were aligned with the overall goal of the program, confirming the program's appropriateness for this context. These discussions also corroborate what the survey of migrant women suggest: services are acceptable and appropriate as evidenced by their high utilization.

#### 3.2.3 Enhanced reach but feasibility issues

When taking the operationalized definitions of reach, feasibility, and sustainability together (Table 1), one understands the impact of the program, its limits, and key features of the program that will maintain it into the future.

Qualitative findings support the survey data on women's self-reported travel costs, time, and convenience, specifically in demonstrating the enhanced reach of prenatal and family planning services through the outreach platform. Indeed, this was one of the main aims of initiating the program. Ultimately reaching the target of 16 outreach locations, the program rollout was gradual, adding outreach sites over time—balancing reach and feasibility. Tension was felt between the immense logistical effort to reach communities that were very distant from fixed clinics (where outreach operations were based) and the low numbers of women that may be seen on those visits. The following quote highlights some of these issues around reach and demonstrates how outreach could be limited due to logistical constraints.

The distance is far for [SMRU/BHF staff, Aw Sor Tho]. Many spots do not have many patients. This suggests that in the future that the ones that have few patients potentially be stopped.— Focus group discussion with SMRU/BHF outreach staff, Mae Ramat District.

The SMRU/BHF partnered with M-Fund (migrant health insurance) to provide insurance to migrant women in the region. M-Fund was deemed a possible intervention to offset the potentially catastrophic costs that women face during obstetrical emergencies. M-Fund uptake and enrollment was greatly enhanced by M-Fund staffers working alongside the outreach team and coordinating with Aw Sor Tho.

To work with Aw Sor Tho is a real benefit. Most of the places we have to rely on the Aw Sor Tho because they cannot get up-to-date information on the patients and we need to communicate with the Aw Sor Tho in case something happens. This helps a lot for the outreach clinic staff and M-Fund.— Focus group discussion with M-Fund staff.

M-Fund staff and MCH service should continue. It saves a lot of the pregnant women costs for transportation. When the outreach sites are in their communities they don't need to pay for transportation, police extortion. This also allows them more money to pay for the monthly fees for M-Fund. This is a benefit for both programs and in the community.— Focus group discussion with M-Fund staff.

However, there were unforeseen follow-on effects wherein women unable to meet M-Fund monthly premiums collected on outreach and in fixed clinics would “miss” prenatal and family planning appointments.

M-Fund has some problems between Aw Sor Tho and pregnant women. Pregnant women don't have money for the M-Fund, so the Aw Sor Tho will help by giving their own money. But sometimes they are not paid back— Focus group discussion with Aw Sor Tho/, Phop Phra District.

Similar to reach, the feasibility of the outreach program also met with some limitations. The participants who were highly vocal about feasibility included the SMRU/BHF staff (nurses, midwives, medics, IT, and general support staff) and Aw Sor Tho. The outreach workload was also considerably high and required early preparation, delivery of services during the outreach visit, and follow-up tasks when a visit was completed. The outreach staff provided some examples of the challenges involved in providing services at outreach sites, which suggested limits on the feasibility of the operation:

Ultrasound and vaccines: when we are in the field we have to do all our own work [i.e., alone] because it is very specialized so we cannot always ask for help. But at the fixed clinic there are two trained staff and they can share the tasks.— Focus group discussion with SMRU/BHF outreach staff, Mae Ramat District.

There is a large counseling burden on outreach for so many new interventions and treatments especially for family planning and there is only one counselor.— Focus group discussion with SMRU/BHF outreach staff, Phop Phra District.

Although outreach locations were closer to women, work and mobility could still impede or prevent women from receiving care:

From the information about women provided by the clinic, the health workers call women that are needed to be found. But migrant women change phones very often and that makes it difficult to follow up. We need to know the name of the compound, name of their employer, as well as their name. Without that information it is hard to find women.— Focus group discussion with Aw Sor Tho, Phop Phra District.

Although these may have affected the feasibility of the program, this likely did not impact the overall program success, given the high utilization of services and self-reported satisfaction of the women surveyed.

#### 3.2.4 Program sustainability

The sustainability of this outreach program was also in the forefront of the minds of many participants. Although there were doubts whether such an immense effort on the part of an NGO could be sustained in the long term, many participants noted that the program improved logistical and administrative practices within SMRU/BHF, allowed for task shifting to Aw Sor Tho, and offloaded the reproductive care of undocumented migrants provided by the Thai public health system.

The sustainability of the program was attributed to a strong community engagement approach that incorporated the capacity building of Aw Sor Tho. Prior to piloting outreach across the two districts in Tak Province, Thailand, SMRU/BHF performed needs and community assessments identifying stakeholders in the Thai, NGO, and CBO sectors of Tak Province providing care to migrant women. The initial part of the pilot phase invested in engaging Tak provincial and district-level public health officials (44) to receive feedback on the outreach program design and apprise officials of the proposed scope of work by SMRU/BHF in these districts. Community engagement, led by a specialized team at SMRU/BHF, assessed and identified possible locations for outreach services. In addition, these engagement activities provided the initial contact for many migrant communities about the scope and aims of the proposed outreach program, soliciting feedback, identifying local partners and Aw Sor Tho, and enhancing community acceptance for the proposed outreach program.

There was community engagement prior to the program beginning. This included high level [Thai system] and local level (communities) that needed to be engaged prior to and during the outreach program. This built a lot of trust for SMRU/BHF among Aw Sor Tho, the Thai system, and communities.— Focus group discussion with SMRU/BHF support staff.

Therefore, by the time the program began, the SMRU/BHF had sensitized several key stakeholders across Thai, NGO, CBO, and local communities around the outreach program.

Training and capacity building of SMRU/BHF staff and Aw Sor Tho allowed for task shifting as a means of sustaining the SMARH-T Initiative. As the pilot phase started, full training activities as part of the outreach program commenced. Training of SMRU/BHF staff involved in the outreach program allowed for capacity building in translating fixed clinic delivery of services to community settings. This training program was proactively adapted and provided gradually to the Aw Sor Tho to increase their understanding of reproductive, maternal, and newborn health.

It's been a big learning curve to have to work with [Aw Sor Tho]. Have to understand how to help improve their capacity. “What can they do?” Now after working with them and the curriculum…it was probably a good first step because it taught us what they were capable of.—Focus group discussion with SMRU/BHF doctors.

The SMARH-T program implemented measures to monitor, refresh, and supervise the tasks of the Aw Sor Tho over the course of the outreach program.

As outreach visits began, SMRU/BHF built on these initial investments, working with the Aw Sor Tho to identify task shifting that may maintain outreach efforts. It also enhanced the capacities of the Aw Sor Tho by drawing from their diverse and often deep experience working with public health initiatives in the region in the past. Rolling out this training was a trial-and-error exercise: it required iterations to effectively match the level and depth of the training to the unique and varied skill sets of the Aw Sor Tho.

Over 90% of all the FP services are run completely by the [SMRU/BHF] staff. They do have questions for [the doctors], but it's really impressive how much they can do— Focus group discussion with SMRU/BHF doctors.

The Aw Sor Tho also mentioned how difficult the training process was, but many reported that they greatly benefited and felt empowered to help their communities. The program included many Aw Sor Tho who were traditional birth attendants who now recommended that women receive services on outreach as opposed to performing services themselves.

Most of us [Aw Sor Tho] are traditional birth attendants, but now we have learned more about the proper procedures. Also, task shifting changed our mind about what is safe to do for ourselves and what has to be referred to SMRU/BHF. Now we rarely do home births by ourselves but instead send the women for delivery at SMRU/BHF fixed clinics.—Focus group discussion with Aw Sor Tho, Phop Phra District.

Community engagement activities feature prominently in ongoing SMRU/BHF efforts, led by Aw Sor Tho, who have gained knowledge and confidence through SMRU/BHF training. These community health worker–led engagement activities were expanded in frequency and reach over the entire course of the initiative.

Engagement at all levels of the health system was made possible only by institutionalizing general program support staff at the SMRU/BHF, who are known as “bridge persons.” These general outreach program support staff often filled gaps in training, community engagement, and advocacy and improved the working relationship between SMRU/BHF medical staff and Aw Sor Tho by liaising effectively between the two groups.

We were already helping in the community, but now we have joined SMRU/BHF, we have gained more knowledge and confidence especially through being supported from SMRU/BHF support staff [i.e., “bridge persons”]. Before we were helping but only with minimal knowledge and now we are knowledgeable and can give more information to the communities.—Focus group discussion with Aw Sor Tho/, Mae Ramat District.

Finally, successful outcomes of the SMARH-T Initiative were further substantiated by Thai partners who realized the indirect benefits that they received from SMRU/BHF outreach service delivery. Sustainability was reflected by Thai public health participants in a way that their services were now increasingly focused on the populations within their remit: Thai nationals and registered migrants.

The problem with [vaccinations] among migrants is missed appointments, late for vaccine after delivery, or they disappear then come back again when the child is already 1 year. When I was first in charge for vaccine, there were almost 30 children receiving vaccines here. Now there are about 15–20 because of SMRU/BHF performing outreach in the community. Some who live close to the [Thai Health Promoting Hospital] will go there, and those who live close to SMRU/BHF outreach will go to SMRU outreach.—Focus group discussion with Thai health officers, Mae Ramat District.

As such, the qualitative components of the study corroborate many of the findings from the quantitative components while providing the nuances in program implementation for the factors influencing reach, feasibility, and sustainability.

## 4 Discussion

This study used a mixed-methods approach to evaluate a successful maternal, newborn and child health program for migrant women along the Thailand–Myanmar border. The quantitative findings and high utilization of services by women from these marginalized communities substantiated the logic model (Figure 1) of delivering services through a mobile, outreach platform supported by fixed clinics for prenatal, family planning, and delivery care. Women who were surveyed suggested that the outreach delivery of acceptable and appropriate maternal, newborn, and child health services offset the structural barriers that many women face in receiving quality care in this region. The qualitative methods in this study captured the voices of providers, community health workers/Aw Sor Tho, Thai health officers, and other stakeholders associated with the program who provided evidence that the outreach program design offset the considerable structural determinants preventing timely care for women in their communities. Participants explained the study findings of high utilization due to service delivery within migrant communities and cited end user satisfaction, acceptability, and appropriateness of the program among the served migrant women. This study provides real-world evidence for a package of evidence-based clinical and community interventions (3, 10, 45) implemented in an LMIC setting. It suggests a mechanism for how this program works, the inputs needed, and the outputs observed–adapted to the local context (Figure 1) (46).

Although beginning in 2020 ahead of the Lancet series, “Maternal Health in the Perinatal Period and Beyond,” which encompasses prenatal and postpartum family planning care delivery, the SMARH-T program aligns well with this series, developing a platform for delivery of care that offsets the structural and social determinants facing this population. The Lancet series approaches maternal health with an eye to vulnerability, intersectionality, risk of poor health outcomes, and potential ways of “repair”—i.e., areas to intervene to reduce risk (6, 47). This work summarized important theoretical and practice contributions of authors looking at intersectionality, vulnerability, and their impact on maternal health (47–51). Importantly, this series informs conceptualizing vulnerability as fluctuating over the course of the prenatal and postnatal period, as opposed to being viewed as a binary variable that remains static and unchanged from the moment of conception. Researchers and public health practitioners are now afforded better ways to think through and implement equity-informed interventions across the prenatal and postnatal course that offsets risk with timely and appropriate methods of repair (6, 46, 52, 53).

As such, the SMARH-T Initiative is a strong example of incorporating intersectionality and vulnerability specific to these communities and addressing these through sensitive, flexible, and responsive programming for prenatal care and family planning services. This program takes from conceptual notions of intersectionality and vulnerability (6, 48–51), and created a multi-pronged approach that adapts these concepts to local realities for effective service delivery (7, 12, 46, 47, 52, 53). Many of the SMARH-T community-based activities have worked and been proven cost-effective in other LMIC settings to reduce maternal mortality and morbidity due to pregnancy complications (54–57).

This SMARH-T Initiative on the Thailand–Myanmar border draws parallels to the determinants of reproductive health access in other vulnerable populations around the world. Such populations include refugees, migrants, and forcibly displaced persons in LMICs. Time and again it has been demonstrated that women in these situations experience the same structural barriers seen along the Thailand–Myanmar border: geographical barriers; transportation issues; financial limitations; awareness of where to access services; stigma; gender inequity in decision-making; physical and political insecurity (58–64). We are continuing to learn how cross-border communities face disrupted access to prenatal care and family planning and how these are exacerbated by crises such as the COVID-19 pandemic, political and civil conflict, or both (65, 66). Hence, the SMARH-T Initiative may be instructive for providing culturally sensitive care to Myanmar migrant women in other parts of Thailand (67).

In summarizing what was known about this population over many decades of research, three key barriers faced by migrant women were identified and targeted through implementation strategies used in this program to enhance care access: political insecurity, geographic barriers, and cost. As reported in the quantitative and qualitative components of the project, women reported greater convenience in accessing outreach sites, costs similar to women living closer to fixed clinics, and similar levels of satisfaction with the care received on outreach compared to fixed clinics. These findings were rendered possible by the focus on the lived experience of migrant women, and the SMARH-T Initiative provided the flexibility needed to maintain service continuity in times of crisis such as the COVID-19 pandemic and the Myanmar coup. The participants highlighted the immense effort that this program was required to make, with substantial gains achieved in the areas of maternal, newborn, and child health in this population.

### 4.1 Strengths and limitations

This study is not without limitations. Although it would have been useful to better align the prenatal and family planning surveys, logistically this was difficult as family planning consultations finish more quickly than prenatal care visits. The factors of social desirability bias, selection bias, and outreach site representativeness may have limited the findings in the surveys in this study. However, the cross-sectional findings were explained by the qualitative portion of the study, making for more robust assertions about the benefits of the outreach program. This study was not sufficiently long enough—nor was it within this study's scope—to effectively determine the program's effect on population health indicators such as maternal mortality, near miss maternal morbidity cases, and newborn, infant, and under-5 child health outcomes. We note preliminarily that maternal mortality has greatly decreased compared with historical data and assume that there are likely follow-on effects that reduce adverse outcomes in maternal, neonatal, infant, and child mortality. However, this comes with a caveat that maternal mortality ratios recorded for the outreach program may not be directly comparable to historical data. Ongoing implementation of the outreach program will allow for creating more robust, longitudinal data for key population health indicators of interest.

The next iteration of SMARH-T will focus on integrating care for migrant women in the Thai health system. This is important given the continued conflict in Myanmar and reliance on migrant labor in Thailand and limited external funding avenues worldwide. For exploring how this model can be adapted elsewhere and for enhancing its generalizability, future research opportunities should include cost modeling, or a prospective cost analysis should be added to future iterations of this program to inform local-, provincial-, national-, and regional-level policymakers. There are opportunities to further characterize the inputs for this program, including the elements of implementation mapping that also include behavioral determinants and performance/task objectives for specific program staff and stakeholders (70). These implementation mapping and planning techniques can also focus more on gaps in service delivery, such as for long-acting reversible contraceptive utilization.

## Data Availability

The raw data supporting the conclusions of this article will be made available by the authors, upon request.
