# Supplementary material for: A mixed-methods evaluation of outreach service provision by the “Strengthening Migrant Access to Reproductive Health in Thailand” Initiative, 2020–2024
Source: Front Glob Womens Health. 2026 Apr 10;7:1637785. doi: 10.3389/fgwh.2026.1637785 (PMC13106189; doi:10.3389/fgwh.2026.1637785)
Supplement: Supplementary file 1 [file Table1.docx]

Supplemental table 1. Comprehensive package of reproductive health services offered by SMRU/BHF (adapted from the WHO (4,5), Vaivada (3), and Hashmi (16)).

| Preconception and pregnancy | Labor, birth, newborn^a^ | Interventions from preconception to newborn care | Family planning | Community Health Workers (Aw So Tho) roles |
| --- | --- | --- | --- | --- |
| Routine antenatal care, management of maternal chronic illness and pregnancy complications 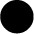 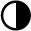 Maternal nutritional supplementation^b^  Lifestyle interventions for gestational diabetes mellitus^c^ 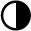 Prenatal infection screening and treatment 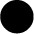 Periconceptional folic acid^d^ 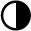 Small quantity lipid-nutrient supplements 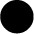 Support for maternal mental health 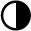 Screening for gender-based violence 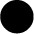 | Skilled birth attendance 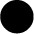 Clean birth kits 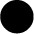 Delayed cord clamping and hygienic cord care 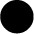 Emergency management of birth complications and asphyxia 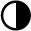 Kangaroo mother care for healthy and low birthweight neonates^e^ 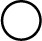 Corticosteeroids for imminent preterm birth, specialized care for preterm, low birthweight, and ill neonates 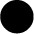 Promotion of early initiation and exclusive breastfeeding 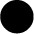 Maternal antibiotics for prolonged premature rupture of membranes 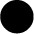 Topical emollient therapy for preterm neonates 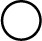 Ibuprofen for patent ductus arteriosus^f^ 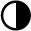 Magnesium sulfate for fetal neuroprotection and early development intervention for preterm infants^g^ 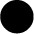 Sound reduction for preterms in NICU  Prophylactic phototherapy for preterm birth and low birthweight neonates with jaundice 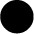 | Routine age-appropriate vaccination for mothers 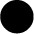 Routine age-appropriate vaccination for infants^h^ 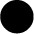 Provision and promotion of insecticide-treated bednets 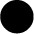 Antibiotic treatment for severe infections and sepsis 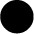 Promotion of improved water, sanitation, and hygiene conditions 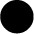 Prevention of gender-based violence 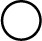 | Combined oral contraceptives 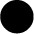 Monthly injectables 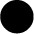 / 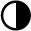 / 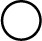 Progestin-only pills 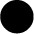 / 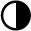 / 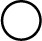 Progestin-only injectables 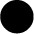 Implants 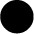 / 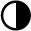 / 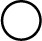 Cu- and LNG-IUDs 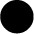 Male and female condoms 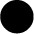 / 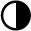 / 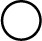 Diaphragms and cervical caps 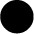 / 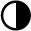 / 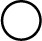 Spermicides 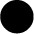 / 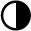 / 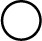 Female sterilization 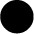 / 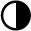 / 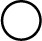 Vasectomy 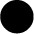 | Community engagement 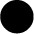 Health Education and Promotion 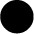 Recruitment 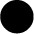 Follow up 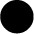 Registration 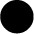 Vital Signs 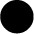 Pregnancy Testing 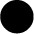 |

= full coverage;
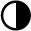
 = partial coverage;
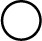
 = no coverage.


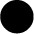


^a^ Care provided at fixed clinics, only.

^b^ Provided as micronutrient-fortified foods and food supplements at prenatal care visits.

^c^ Risk factor-based screening for gestational diabetes mellitus with treatment over the antenatal period (Gilder 2014).

^d^ Periconceptional folic acid coverage is low (<2%) (Stevens).

^e^ Culturally adapted swaddling (White 2012)

^f^ Not all murmurs are routinely checked with cardiac ultrasound.

^g^ Given to mothers at risk of pre-eclampsia.

^h^ Vaccination program in place and provides infant immunizations, but poor follow up restricts coverage rates in the first year of life (Gilder 2022, Bierhoff 2019).
